# Sodium Houttuybonate Promotes the Browning of White Adipose Tissue by Inhibiting Ferroptosis via the AMPK-NRF2-HO1 Pathway

**DOI:** 10.3390/antiox13091057

**Published:** 2024-08-30

**Authors:** Wenhui Liu, Huren Zou, Danming You, Huijie Zhang, Lingling Xu

**Affiliations:** 1 Department of Endocrinology, Shenzhen Hospital, Southern Medical University, Shenzhen 518000, China; 2 Department of Endocrinology and Metabolism, Nanfang Hospital, Southern Medical University, Guangzhou 510000, China

**Keywords:** *houttuynia cordata*, sodium houttuyfonate, iWAT, browning, ferroptosis, obesity

## Abstract

The rising prevalence of obesity has resulted in an increased demand for innovative and effective treatment strategies. *Houttuynia cordata* Thunb. *(H. cordata)* has demonstrated promising potential in preventing obesity. However, the mechanism underlying the anti-obesity effects of *H. cordata* and its bioactive component, sodium houttuybonate (SH), remains unclear. Our study reveals that SH treatment promotes the browning of inguinal white adipose tissue (iWAT) and prevents the obesity induced by a high-fat diet. SH significantly mitigates ferroptosis by upregulating glutathione peroxidase 4 (*Gpx4*) and decreasing malondialdehyde (MDA) levels, while also enhancing superoxide dismutase (SOD) levels. Furthermore, SH promotes the phosphorylation of AMP-activated protein kinase (AMPK), which subsequently increases the expression of nuclear factor erythroid 2-related factor 2 (NRF2) and heme oxygenase-1 (HO-1) in the iWAT. However, the effects of SH were attenuated by ML385, an Nrf2 inhibitor. Collectively, our findings suggest that SH induces iWAT browning and prevents diet-induced obesity primarily through the AMPK/NRF2/HO-1 pathway by inhibiting ferroptosis.

## 1. Introduction

Obesity has emerged as a significant public health challenge, substantially increasing the risk of cardiovascular diseases [1], diabetes [2], non-alcoholic fatty liver disease [3], and specific cancers [4,5]. The primary driver of obesity is the dysfunction of adipose tissue, with disruptions in its triglyceride (TG) storage capacity contributing to glucose dysregulation and associated metabolic complications [6]. The emergence of beige adipocytes within white adipose tissue (WAT), a process known as ‘browning’, facilitates energy dissipation through thermogenesis, predominantly mediated by uncoupling protein 1 (UCP1) [7]. This browning process is recognized for its potential to counteract obesity [8], leading to increased clinical interest in therapeutic strategies that target WAT browning to address obesity and related metabolic disorders.

**Figure 1:**
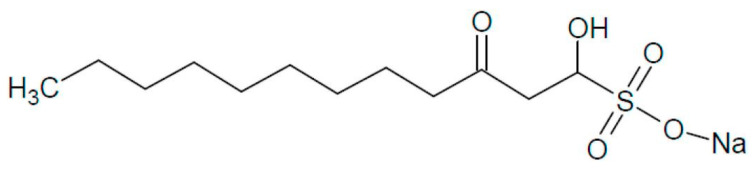
The chemical structure of sodium houttuyfonate (C12H23NaO5S, PubChem CID: 23663544, CAS: 83766-73-8) with a molecular weight (MW) of 302.36 g/mol.

*Houttuynia cordata* Thunb (*H. cordata*), a plant abundant in China and renowned for its medicinal properties, including diuretic and detoxifying effects, is also classified as a “medicine-food” herb, containing over 220 identified compounds [9,10]. These compounds include flavonoids, volatile oils, phenolic acids, alkaloids, terpenes, phenylethanoid glycosides, and phenylpropanoids, with flavonoids and volatile oils serving as the primary active components [11]. Sodium houttuyfonate (SH, Figure 1), a major bioactive compound found in the volatile oil of *H. cordata*, has been noted for its anti-inflammatory [12], antiviral [9], and antidiabetic attributes [13]. Previous studies have suggested that the leaves of *H. cordata* may combat obesity by inhibiting the uptake of non-esterified fatty acids (NEFAs) and glycerol in the small intestine [14], as well as by reducing lipogenesis in mature 3T3-L1 adipocytes through enhanced lipolysis [15]. Nonetheless, the specific mechanisms underlying SH’s anti-obesity effects, particularly whether it promotes the browning of the iWAT, remain to be fully clarified.

Therefore, our research aims to elucidate the mechanisms through which SH exerts its anti-obesity effects, specially focusing on the induction of iWAT browning. This investigation seeks to highlight the potential of SH as an innovative therapeutic strategy for the treatment of obesity.

## 2. Materials and Methods

### 2.1. Mice and Treatments

Male C57BL/6J mice, aged 8–10 weeks, were purchased from Guangdong Zhiyuan Biopharmaceutical Technology Co., Ltd. (Guangzhou, China). The mice were kept at 22 °C at room temperature under a 12 h light/dark cycle with free access to food and water. All the animal procedures were performed in accordance with the procedures approved by the Ethics Committee of Animal Research of Southern Medical University.

The mice were grouped 5–6 per cage and fed a high-fat diet (HFD; 60% kcal from fat, D12492; research diets; New Brunswick, NJ, USA) for 12 weeks to induce obesity. Concurrently, these mice were divided into two subgroups: one received water with a vehicle and the other received water supplemented with 5% SH. Their body weights were recorded weekly to evaluate the effect of the SH on the progression of obesity.

The C57BL/6J mice were randomly assigned to either a vehicle group or an SH treatment group (50 mg/kg, intraperitoneal injection) for 3 days, followed by cold exposure (at 6 °C) for 4 h. Additionally, another set of C57BL/6J mice was randomly allocated to receive either a vehicle, SH, the Nrf2 inhibitor ML385 (50 mg/kg, intraperitoneal injection, Topscience, Shanghai, China), or a combination of ML385 and SH for 3 days, followed by cold exposure (at 6 °C) for 4 h.

In addition, the C57BL/6J mice were randomly allocated to either a vehicle group or an SH treatment group, receiving 50 mg/kg of SH via intraperitoneal injection for 3 days at 30 °C in a temperature-controlled chamber. Each mouse was housed separately with unrestricted access to water and food, allowing the observation of SH’s effects on physiological responses under varying temperature conditions.

### 2.2. Glucose Tolerance Tests (GTTs)

The mice were fed an HFD for 12 weeks, before the GTTs were performed in the 11th week. The mice were fasted overnight before receiving an intraperitoneal injection of D-glucose at a dosage of 1 g/kg body weight. Their blood glucose levels were measured using a glucose meter (ACCU-CHEK, Roche, Basel, Switzerland) by puncturing the tail vein at 0, 30, 60, 90, and 120 min post-injection.

### 2.3. Metabolic and Biochemical Analyses

The serum biochemical parameters, including triacylglycerol (TG), aspartate aminotransferase (AST), and alanine aminotransferase (ALT), were measured according to the manufacturer’s instructions (Nanjing Jiancheng Bio, Nangjing, China). The concentrations of malondialdehyde (MDA), reduced glutathione (GSH), and superoxide dismutase (SOD) in both the serum and stromal vascular fraction (SVF) cell lysate were also assessed according to the protocols provided by Beyotime Bio (Shanghai, China).

### 2.4. Histological and Immunohistochemical Analyses

The adipose tissues were fixed in 10% formalin, before being subsequently dehydrated, embedded in paraffin, and sectioned. For the histological analysis, the sections were deparaffinized and stained with hematoxylin and eosin. For the immunohistochemical (IHC) staining, endogenous peroxidase was blocked and antigen retrieval was applied. After the blocking, the sections were incubated overnight at 4 °C with primary antibodies against UCP1 (1:500, Proteintech, Wuhan, China) and NRF2 (1:100, Abclonal, Wuhan, China). The sections were then incubated with a goat anti-IgG HRP secondary antibody (1:1000, Abcam, Cambridge, UK) and developed with 3-amino-9-ethylcarbazole (AEC), followed by counterstaining with hematoxylin. The images were captured using an inverted microscope at a magnification of ×200, with 5 fields imaged per slide. The UCP1 and NRF2 staining were analyzed by measuring the positive area and calculating the percentage of the positive area using ImageJ software 1.8.0.

### 2.5. Primary SVF Isolation, Maturation, and Treatment

Inguinal fat pads from 3- to 5-week-old male mice were dissected, washed, and then digested for 30 min at 37 °C in PBS containing 10 mM of CaCl_2_ and 3 mg/mL of collagenase. The digested tissues were then filtered through a 40 μm cell strainer and centrifuged at 1000× *g* for 10 min to pellet the stromal vascular cells. The cells were re-suspended in DMEM-F12 medium (Gibco, Carlsbad, CA, USA) until reaching confluence, after which adipocyte differentiation was induced using a standard cocktail (DMEM/F12 containing 10% FBS (Excell, Suzhou, China), 0.5 mM of isobutylmethylxanthine, 62.5 μM of indomethacin, 2 μg/mL of dexamethasone (Sigma-Aldrich, St. Louis, MO, USA), 1 μM of insulin (Sigma-Aldrich, St. Louis, MO, USA), 1 nM of T3 (Sigma-Aldrich, St. Louis, MO, USA), and 1 μM of rosiglitazone (Sigma-Aldrich, St. Louis, MO, USA)). After 48 h, the cells were switched to a maintenance medium (DMEM/F12 containing 10% FBS, 1 μM of insulin, 1 nM of T3, and 1 μM of rosiglitazone).

The SH was administered at a concentration of 50 µg/mL during the differentiation process for 1, 3, and 7 days, respectively. Additionally, the cells were treated with RSL3 (10 µM) or ML385 (10 µM) for 48 or 72 h, respectively.

### 2.6. Quantitative PCR (qPCR) Analysis

The total RNA was extracted from the cells or adipose tissues using TRIZOL reagent (ThermoFisher, Waltham, MA, USA), following the manufacturer’s instructions. The extracted RNA was subsequently reverse-transcribed into cDNA with the 5× Evo M-MLV RT Master Mix (AG, Wuhan, China). A quantitative real-time PCR was performed using the SYBR Real-time PCR Master Mix kit (Yeason, Shanghai, China) on the LightCycler480 (Roche, Basel, Switzerland). The data obtained from the quantitative real-time PCR were analyzed using the 2^−ΔΔCt^) method. The primers used are listed in Table S1.

### 2.7. Western Blotting

The SVF cells were lysed with RIPA buffer (Beyotime, Shanghai, China) containing a protease inhibitor cocktail and a phosphatase inhibitor cocktail on ice, and the supernatants were harvested. The total lysate protein was boiled with loading buffer and then separated on SDS-PAGE gels and transferred to PVDF membranes (Millipore, Billerica, MA, USA). The membranes were blocked with an efficient Western Blot quick-sealing liquid (Genefist, Guangzhou, China) and then incubated overnight at 4 °C with the indicated primary antibodies, followed by incubation with the secondary antibodies for 1 h at room temperature. The protein expression was detected using the Tanon Chemiluminescent Imaging System (Tanon 5200 Multi). The specific antibodies used are listed in Table S2.

### 2.8. Data Statistics

All data analyses were analyzed using GraphPad Prism 6. The data are presented as the mean ± standard error of the mean (SEM). The statistical significance between the two groups was determined using an unpaired two-tailed Student’s *t*-test, while the differences among more than two groups were assessed using a two-way analysis of variance (ANOVA) followed by multiple comparisons. A *p*-value of <0.05 was considered statistically significant.

### 2.9. Data and Resource Availability

All the data generated during this study are included in this published article (and the Supplementary Material).

## 3. Results

### 3.1. SH Promotes iWAT Browning and Prevents Diet-Induced Obesity

**Figure 2:**
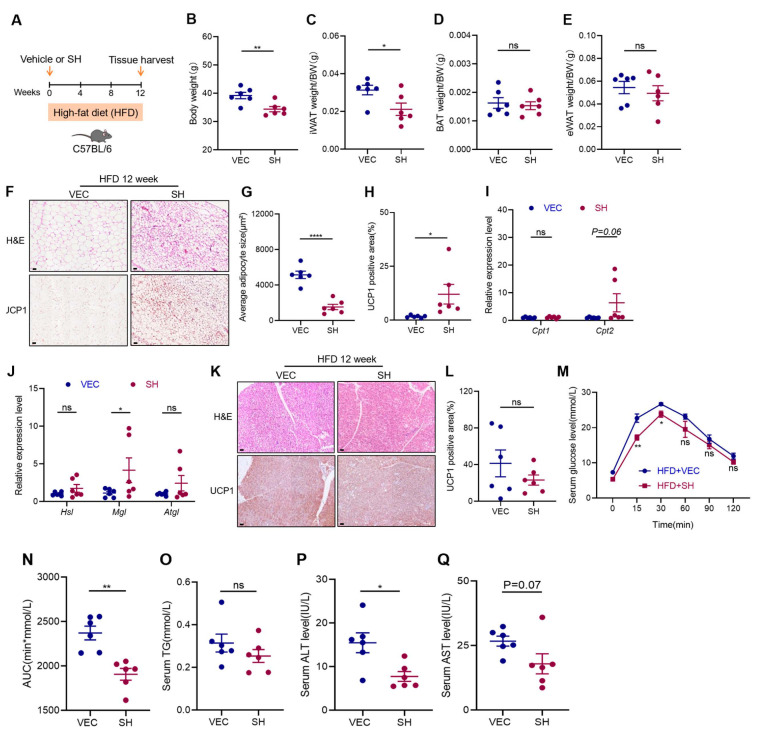
SH promotes the browning of the iWAT in HFD-induced obese mice. (**A**) Experimental design for the treatment of SH during a 12-week high-fat diet (HFD) feeding experiment. (**B**) Body weight during the 12-week intervention with SH. (**C**–**E**) Post-intervention weights of iWAT, BAT, and eWAT relative to body weight in both the vehicle (VEC) and SH-treated mice. (**F**–**H**) Histopathological assessment of the iWAT using H&E staining, including the quantitative analysis of the lipid droplet size and the UCP1^+^ areas detected by the IHC. Scar bar, 120 µm. (**I**,**J**) Relative mRNA expression levels of *Cpt1*, *Cpt2*, *Hsl*, *Mgl*, and *Atgl* in the iWAT. (**K**) Representative H&E and UCP1^+^ areas of the BAT from vehicle-treated (n = 5) and SH-treated (n = 6) mice. (**L**) Quantification of UCP1^+^ areas in BAT. Scar bar, 120 µm.(**M**,**N**) GTTs were conducted after 11 weeks of treatment, with the area under the curve (AUC) calculated for each group. (**O**–**Q**) Serum levels of TG, ALT, and AST. All data are represented as mean ± SEM. * *p* < 0.05, ** *p* < 0.01, and **** *p* < 0.0001.

To investigate the mechanism underlying the anti-obesity effects of SH, 8-week-old male C57BL/6J mice were administered SH while being fed an HFD for 12 weeks (Figure 2A). The mice treated with the SH exhibited greater weight loss and reduced iWAT mass compared to the control mice, with no significant changes in the BAT or epididymal white adipose tissue (eWAT) observed (Figure 2B–E). The H&E staining of the iWAT from the SH-treated groups revealed smaller adipocyte sizes and increased multilocular lipid droplets (Figure 2F,G). Furthermore, the SH treatment resulted in the increased expression of UCP1 in the iWAT (Figure 2H), which aligns with the enhanced fatty acid oxidation and lipolysis observed after 12 weeks of SH exposure (Figure 2I,J). In contrast, no significant difference in the thermogenic effects of the BAT was observed (Figure 2K,L). Additionally, the SH-treated mice exhibited a significantly improved glucose tolerance compared to the controls (Figure 2M,N). To assess the potential toxicity, the serum levels of ALT and AST were measured in both the vehicle and SH-treated mice. The results indicated that the SH treatment led to reduced ALT levels and slightly lower AST levels compared to the vehicle controls under HFD conditions (Figure 2P,Q). These findings suggest that SH promotes the browning of the iWAT and may represent a safe and effective therapeutic strategy against diet-induced obesity.

### 3.2. Effects of SH on Adipose Tissue under Cold Stimulation

**Figure 3:**
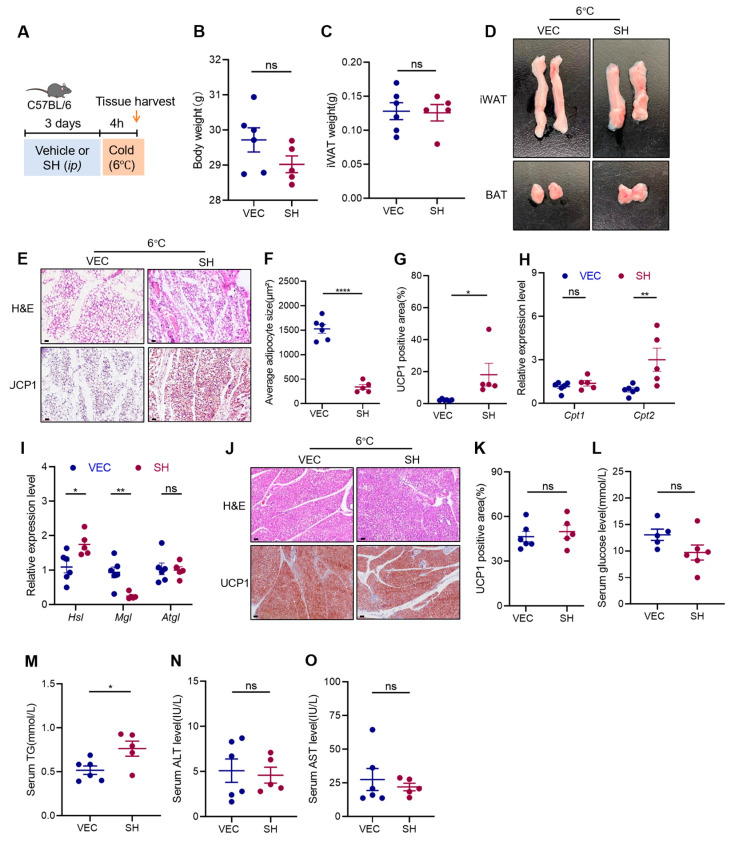
SH promotes the browning of the iWAT in mice exposed to cold stimulation. (**A**) Experimental design for SH treatment in mice exposed to cold. (**B**,**C**) Body weight and iWAT mass of mice after 4 h of cold exposure at 6 °C. (**D**) Morphology of the iWAT and BAT in the vehicle group (n = 6) and SH-treated (n = 5) group at 6 °C. (**E**) Representative images of the iWAT stained with H&E and the UCP1^+^ areas in the iWAT from the vehicle (n = 6) and SH-treated mice (n = 5). Scar bar, 120 µm. (**F**,**G**) Quantification of adipocyte area and UCP1^+^ areas in the iWAT. (**H**,**I**) Relative mRNA expression levels of *Cpt1*, *Cpt2*, *Hsl*, *Mgl*, and *Atgl*. (**J**) Representative images of BAT stained with H&E and the UCP1^+^ areas in the BAT from the vehicle (n = 6) and SH-treated mice (n = 5). Magnification, ×200. Scar bar, 120 µm. (**K**) Quantification of UCP1^+^ areas in BAT. (**L**–**O**) Serum levels of glucose, TG, ALT, and AST. All data are represented as mean ± SEM. * *p* < 0.05, ** *p* < 0.01, and **** *p* < 0.0001.

To further investigate whether SH induces the browning of the adipose tissue, the mice were subjected to cold exposure at 6 °C for four hours following three days of intraperitoneal injections of either the vehicle or SH (Figure 3A). Cold exposure is known to activate the β-3 adrenergic receptors in adipose tissue, promoting non-shivering thermogenesis, increasing lipolysis, and inducing the expression of thermogenic genes such as UCP1. This process, known as the browning of white adipose tissue (WAT), leads to the formation of beige cells. Although there were no significant differences in the mass of the iWAT between the control and SH-treated groups (Figure 3B,C), the iWAT from the SH-treated mice displayed a browner color and a reduced proportion of multilocular lipid droplets (Figure 3D,E). Additionally, the SH-treated mice showed an increased expression of UCP1 in the iWAT, along with enhanced fatty acid oxidation and lipolysis (Figure 3E–I). However, the SH did not significantly affect the morphology or thermogenic activity in the BAT (Figure 3J,K). The SH-treated mice also showed lower blood glucose levels (Figure 3L) and higher triglyceride levels (Figure 3M). Importantly, the SH treatment did not alter the ALT and AST levels (Figure 3N,O), indicating no adverse effects on liver function. Collectively, these results suggest that SH specifically induces the browning of the iWAT, but not the BAT, in response to cold stimulation.

### 3.3. Effects of SH on Adipose Tissue at Thermoneutrality

**Figure 4:**
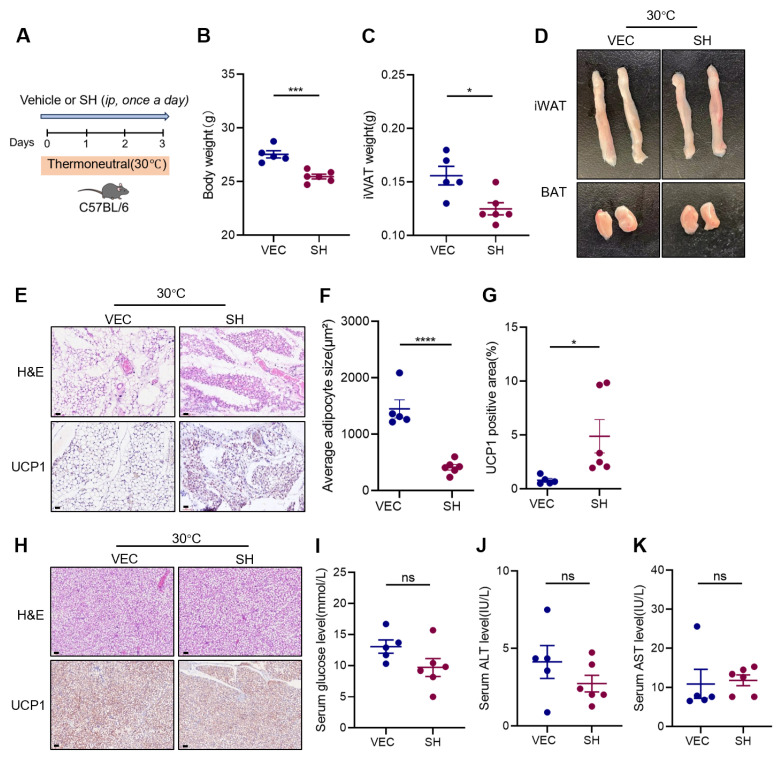
SH promotes the browning of the white adipose tissue in mice at thermoneutrality. (**A**) Experimental design for SH treatment in mice kept at thermoneutral conditions (30 °C). (**B**,**C**) Body weight and iWAT mass following a 3-day intervention with SH under thermoneutral conditions at 30 °C. (**D**) Representative morphology of the iWAT and BAT in the vehicle group (n = 5) and SH-treated group (n = 6) at 30 °C. (**E**) Representative images of the iWAT stained with H&E and the UCP1^+^ areas in the iWAT from the vehicle (n = 5) and SH-treated mice (n = 6). Magnification, ×200. Scar bar, 120 µm. (**F**,**G**) Quantification of adipocyte area and UCP1^+^ areas in the iWAT. (**H**) Representative images of the BAT stained with H&E and the UCP1^+^ areas in the BAT from the vehicle group (n = 5) and SH-treated group (n = 6). Magnification, x200. Scar bar, 120 µm. (**I**–**K**) Serum levels of glucose, ALT, and AST. All data are represented as mean ± SEM. * *p* < 0.05, *** *p* < 0.001, and **** *p* < 0.0001.

At a thermoneutral temperature (30 °C), the brown adipocytes in both mice and humans can transition to a white phenotype. This transition results in a suppression of heat production and a decrease in browning capacity [16]. To examine the effect of SH on the browning of the iWAT under thermoneutral conditions, the male mice were treated with SH for three days, while the temperature was maintained at 30 °C (Figure 4A). The SH-treated mice exhibited significant reductions in both body weight and iWAT mass (Figure 4B,C). Notably, the iWAT of the SH-treated mice displayed a brownish color (Figure 4D), and contained ‘brown-like’ adipocytes characterized by smaller, multilocular lipid droplets (Figure 4E,F). Furthermore, the IHC staining for UCP1 in the iWAT showed an increased expression in the SH-treated mice (Figure 4G). In contrast, no differences were observed in the BAT between the two groups (Figure 4H). Although the SH administration resulted in a slight decrease in blood glucose levels, the serum ALT and AST levels remained unchanged (Figure 4I–K).

### 3.4. SH Directly Promotes the Browning of Adipocytes In Vitro

**Figure 5:**
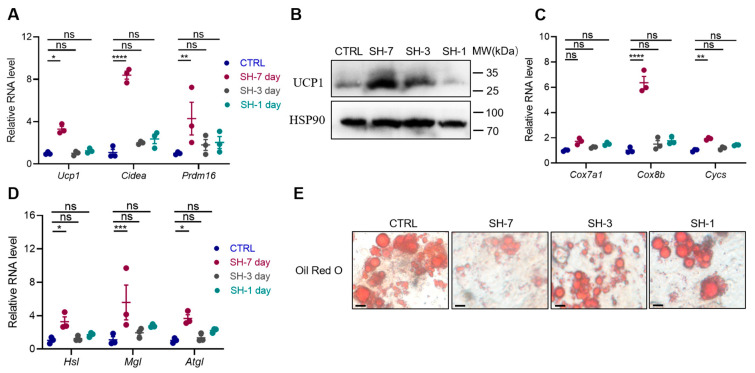
SH stimulates the browning of primary adipocytes. (**A**) The gene expression profiles related to thermogenesis in the primary inguinal adipocytes after treatment with SH. (**B**) UCP1 protein expression levels following treatment with 50 μg/mL of SH for 7, 3, and 1 days. (**C**,**D**) Gene expression profiles related to mitochondrial function and lipolysis in the primary inguinal adipocytes after treatment with SH. (**E**) Representative Oil Red O staining of lipid droplets in adipocytes, showing lipid accumulation following SH treatment. Scar bar, 50 µm. All data are represented as mean ± SEM. * *p* < 0.05, ** *p* < 0.01, *** *p* < 0.001, and **** *p* < 0.0001.

To investigate the direct effects of SH on the browning of the iWAT, an in vitro study was conducted using stromal vascular fraction (SVF) cells isolated from mouse iWAT. A significant increase in the expression of thermogenic genes, including Ucp1, Cidea, and Prdm16, was observed following a 7-day treatment with SH (Figure 5A,B). Additionally, the SH markedly upregulated the expression of mitochondrial-related genes such as Cox7a1, Cox8b, and Cycs (Figure 5C), which are crucial for cellular respiration and energy production. Furthermore, the expression of lipolysis-associated genes, including Hsl, Mgl, and Atgl, was also increased (Figure 5D). These molecular changes corresponded with a reduction in the number and size of lipid droplets, as evidenced by the Oil Red O staining (Figure 5E). Collectively, these results suggest that SH directly induces the browning of the primary adipocytes.

### 3.5. SH Enhances Adipocyte Browning by Inhibiting Ferroptosis

**Figure 6:**
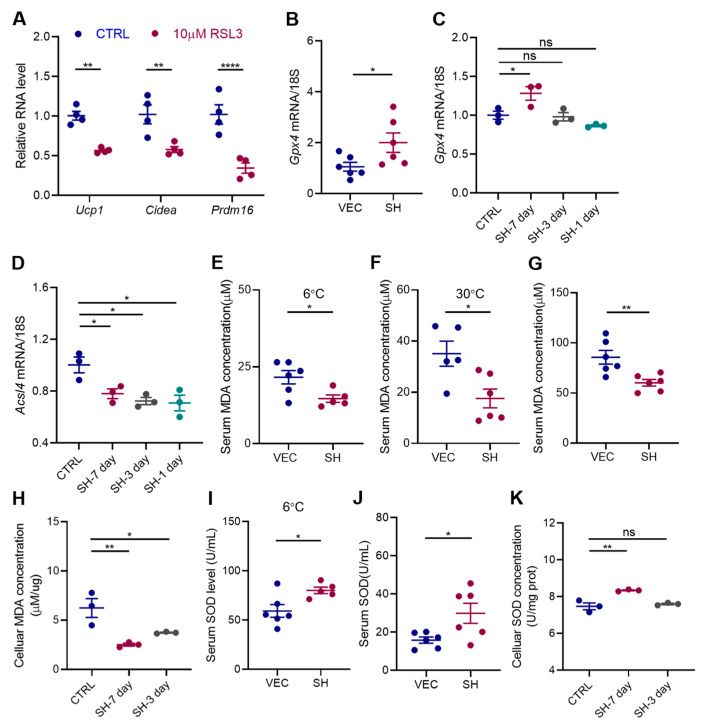
SH impedes ferroptosis under different experimental conditions. (**A**) Relative mRNA expression levels of thermogenic genes in the primary adipocytes treated with 10 μM of RSL3 for 48h. (**B**) Relative mRNA expression of *Gpx4* in the vehicle and SH-treated mice after a 12-week HFD feeding period. (**C**,**D**) Relative mRNA expression of *Gpx4* and *Acsl4* in the primary adipocytes following treatment with 50 µg/mL of SH for varying durations. (**E**–**G**) Serum MDA levels in mice subjected to cold stimulation at 6 °C (**E**), maintained at a thermoneutral condition of 30 °C (**F**), and after a 12-week HFD (**G**). (**H**) Intracellular MDA concentrations in the primary adipocytes following treatment with 50 μg/mL of SH for either 3 or 7 days. (**I**,**J**) Serum SOD levels in mice subjected to cold stimulation at 6 °C (**I**) and after a 12-week HFD (**J**). (**K**) Intracellular SOD concentrations in the primary adipocytes following treatment with 50 μg/mL of SH for either 3 or 7 days. All data are represented as mean ± SEM. * *p* < 0.05, ** *p* < 0.01, and **** *p* < 0.0001.

Excessive lipid accumulation is known to significantly elevate the intracellular reactive oxygen species (ROS), which negatively impacts the browning of the adipose tissue. Ferroptosis, a critical form of cell death, is characterized by the disruption of the intracellular antioxidant defenses and a marked accumulation of the ROS within the mitochondria [17]. However, its role in adipocyte browning has been poorly studied. In our study, the treatment with the ferroptosis inducer RSL3 led to a decrease in the expression of the thermogenic genes in the primary adipocytes, suggesting that ferroptosis may inhibit the browning of the iWAT (Figure 6A). We subsequently investigated whether SH could modulate ferroptosis during browning. Our findings revealed a significant increase in the expression of Gpx4, a key enzyme involved in regulating lipid peroxidation [18], following the SH treatment in both diet-induced obesity (DIO) mouse and primary SVF cells (Figure 6B,C). Conversely, Acsl4, an enzyme responsible for activating long-chain fatty acids [19], was suppressed in the adipocytes treated with SH for 7 days (Figure 6D).

Further analysis revealed that malondialdehyde (MDA), a byproduct of lipid peroxidation and an indicator of ferroptosis, was significantly reduced in the SH-treated mice under both 6 °C and 30 °C conditions (Figure 6E,F), indicating a decrease in oxidative stress. A 12-week SH intervention in the HFD-fed mice also significantly reduced the MDA levels, further supporting the cumulative protective effect of SH against oxidative stress (Figure 6G). Similarly, the MDA levels decreased in primary SVF cells after 7 days of SH treatment (Figure 6H).

Moreover, the levels of superoxide dismutase (SOD), an important antioxidant enzyme that neutralizes superoxide radicals, were also assessed. Consistent with the MDA results, the SOD levels were notably increased in the SH-treated mice exposed to 6 °C and HFD conditions (Figure 6I,J), as well as in the primary SVF cells following a 7-day SH treatment (Figure 6K).

Taken together, these findings indicate that SH effectively enhances antioxidant defenses while reducing lipid peroxidation, thereby inhibiting ferroptosis.

### 3.6. SH Promotes iWAT Browning via the AMPK-NRF2-HO1 Axis

We further elucidate the mechanism by which SH induces browning through the inhibition of ferroptosis. Previous studies have demonstrated that the suppression of erythroid-derived 2-related factor (NRF2) can reverse the ferroptosis resistance commonly observed in response to anti-tumor drug treatments [20]. Additionally, NRF2 deficiency has been shown to accelerate ferroptosis in models of acute lung injury [21]. Considering the role of SH in promoting adipose tissue browning, we hypothesized that SH may enhance iWAT browning by inhibiting ferroptosis via the modulation of the AMPK/NRF2/HO1 pathway.

**Figure 7:**
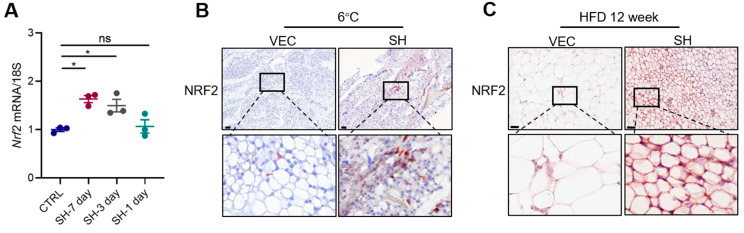

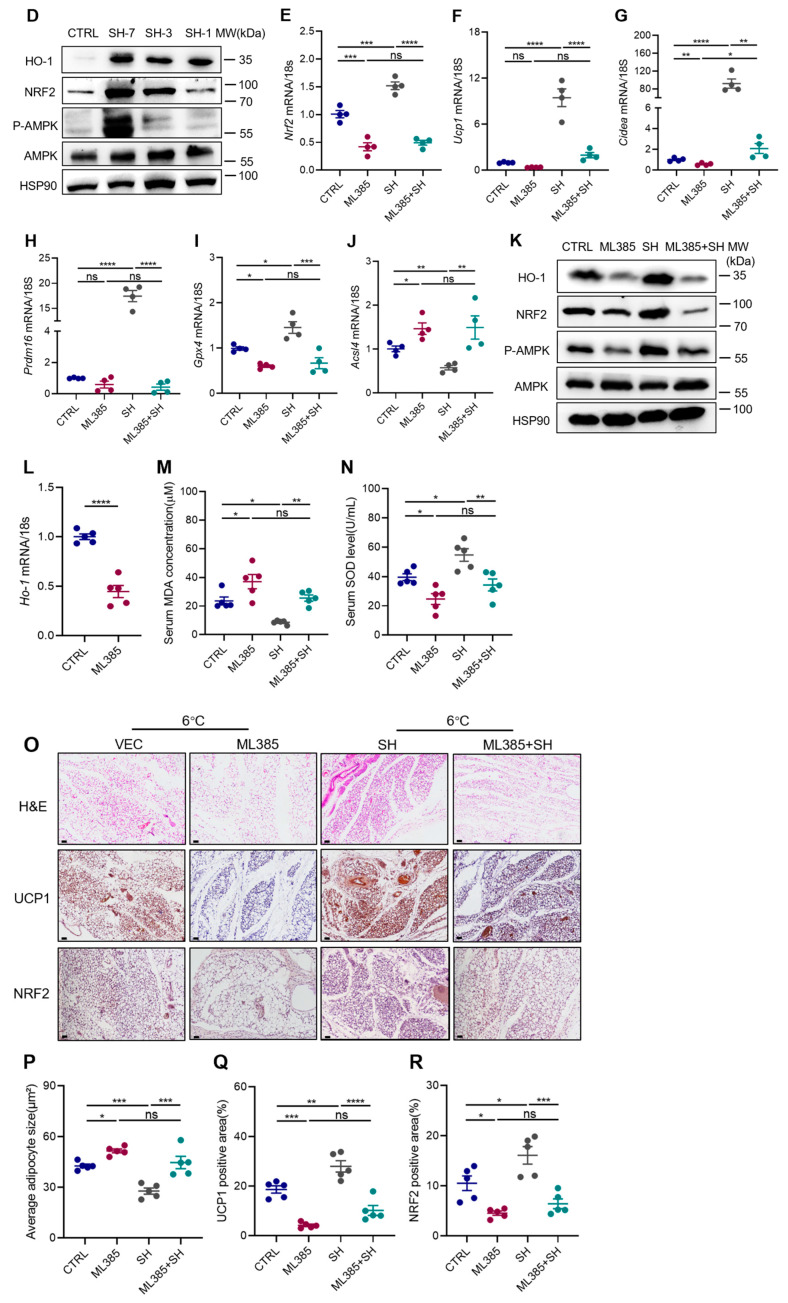
SH inhibits ferroptosis through the AMPK-NRF2-HO1 pathway. (**A**) Relative mRNA expression of *Nrf2* in the primary adipocytes following varying durations of treatment with 50 µg/mL of SH. (**B**) Immunohistochemical staining of Nrf2^+^ areas in the iWAT under 6 °C cold stimulation. Scar bar, 120 µm. (**C**) Immunohistochemical staining of Nrf2^+^ areas in the iWAT under high-fat diet conditions. Scar bar, 60 µm. (**D**) Activation of AMPK/NRF2/HO1 pathway in the primary adipocytes treated with 50 μg/mL of SH. (**E**) Relative mRNA expression of *Nrf2* in the primary adipocytes following treatment with or without 10 µM of ML385 and 50 µg/mL of SH. (**F**–**H**) Relative mRNA expression levels of thermogenic genes in the primary adipocytes treated with or without 10 µM of ML385 and 50 µg/mL of SH. (**I**,**J**) Relative mRNA expression of *Gpx4* and *Acsl4* in the primary adipocytes following treatment with or without 10 µM of ML385 and 50 µg/mL of SH. (**K**) Activation of the AMPK/NRF2/HO1 pathway in the primary adipocytes following treatment with or without 10 µM of ML385 and 50 µg/mL of SH. (**L**) Relative mRNA expression of *Ho-1* was measured in mice treated with or without 20 mg/kg of ML385, followed by cold stimulation at 6 °C. (**M**,**N**) Serum levels of MDA (**M**) and SOD (**N**) in mice treated with either 50 mg/kg of SH or 20 mg/kg of ML385, followed by cold stimulation at 6 °C. (**O**) Representative images of the iWAT stained with H&E, along with UCP1^+^ areas and Nrf2^+^ areas in the iWAT from the vehicle (n = 6) and SH-treated mice (n = 5). Scar bar, 60 µm. (**P**–**R**) Quantification of adipocyte area (**P**) and UCP1^+^ areas (**Q**) and NRF2^+^ areas (**R**). All data are represented as mean ± SEM. * *p* < 0.05, ** *p* < 0.01, *** *p* < 0.001 and **** *p* < 0.0001.

Our results demonstrated a significant upregulation of Nrf2 in both the mRNA and protein levels in the primary adipocytes following the SH treatment, with the most pronounced effect observed after 7 days (Figure 7A,D). The immunohistochemistry results further confirmed that the SH significantly elevated the Nrf2 levels in mouse iWAT under cold stimulation (6 °C) and HFD conditions (Figure 7B,C). The activation of the AMPK/NRF2/HO1 pathway was evident during the SH-induced browning of the primary adipocytes (Figure 7D). These findings suggest that SH may mitigate obesity by promoting browning and inhibiting ferroptosis through the regulation of the AMPK/NRF2/HO1 pathway.

To further confirm the involvement of the AMPK/NRF2/HO1 pathway in the effects induced by SH, the Nrf2 inhibitor ML385 was employed. The treatment with ML385 resulted in a reduction in the expression of thermogenesis-related genes (Figure 7F–H), an exacerbation of ferroptosis (Figure 7I,J), and diminished AMPK phosphorylation (Figure 7K) in both the presence and absence of the SH (Figure 7F–K). Additionally, ML385 increased the serum MDA levels, decreased the SOD levels, reduced browning, and lowered the Nrf2 expression in cold-exposed mice, regardless of the SH treatment (Figure 7L–R). These results highlight the critical role of the AMPK/NRF2/HO1 pathway in mediating the effects of SH in primary adipocytes and mice.

## 4. Discussion

*H. cordata* is widely used as a leafy vegetable and is recognized for its medicinal properties [22], including notable anti-inflammatory [12], antiviral [10]**,** and antidiabetic effects [13]. Additionally, research has indicated that *H. cordata* may combat obesity by disrupting the absorption of fatty acids and glycerol [14]. Nevertheless, there is limited research on the underlying mechanisms of its anti-obesity effects.

The prevention of weight gain is associated with the enhanced browning of the iWAT [23,24]. In this study, we demonstrated that treatment with SH promotes iWAT browning and prevents the obesity induced by an HFD. Our findings revealed a significant upregulation of UCP1 expression, along with a reduction in both the quantity and size of the lipid droplets in the iWAT. These effects were observed under both cold and thermoneutral conditions, as well as in an obesity-inducing mouse model. The reduction in lipid accumulation was accompanied by increased fatty acid oxidation and lipolysis, suggesting that SH modulates adipocyte metabolism to prevent excessive fat accumulation. These observations were further supported by in vitro experiments on primary adipocytes, which revealed the upregulation of genes related to mitochondrial thermogenesis and lipid catabolism.

Ferroptosis is a identified form of programmed cell death [25], characterized by lipid peroxidation and reactive oxygen species (ROS). Previous studies have suggested that excessive ROS may be involved in the mechanism underlying iWAT browning [26,27,28,29,30]. Therefore, we hypothesized that ferroptosis could play a role in the browning of the iWAT. Our results demonstrated that RSL3, a ferroptosis inducer, inhibited iWAT browning. Furthermore, we investigated whether SH promotes the browning of the iWAT by impeding ferroptosis. The results showed a significant reduction in MDA levels, a marked elevation in SOD levels, and reduced expression of ferroptosis-related genes in the SH-treated groups, indicating a decrease in ferroptotic cell death. Therefore, SH could inhibit ferroptosis, thereby promoting the browning of iWAT.

Multiple studies have established the importance of the NRF2-HO-1 axis in regulating ferroptosis [31,32]. Additionally, *H. cordata* has been shown to activate the NRF2-HO-1 signaling cascade, which counteracts intracellular ROS production during lung tumorigenesis [33]. In our study, we confirmed that SH activates the NRF2/HO-1 signaling pathway, contributing to the browning of the iWAT and the inhibition of ferroptosis. Previous research has demonstrated that AMPK and NRF2 signaling work together to maintain cellular homeostasis [34]. We observed a significant increase in AMPK phosphorylation in the iWAT after the SH treatment, along with reduced oxidative damage, which was attenuated by ML385, a NRF2 inhibitor. Thus, the activation of the AMPK-NRF2-HO1 pathway by SH is essential for its anti-obesity effects.

## 5. Conclusions

In summary, this study shows that SH promotes iWAT browning to combat obesity by inhibiting ferroptosis through the AMPK-NRF2-HO1 pathway, highlighting its potential as a natural therapeutic agent. However, the effects are ameliorated when the Nrf2 inhibitor ML385 is used. This study was limited by a lack of further gene knockdown experiments or in vitro cellular validation to clarify the mechanisms underlying SH’s promotion of iWAT browning. Furthermore, additional research involving human clinical trials is essential, along with studies focused on optimizing the dosage and duration of treatment, to further develop SH as a viable pharmacological intervention for obesity.

## Data Availability

The data are contained within this article.

## Supplementary Materials

The following supporting information can be downloaded at: https://www.mdpi.com/article/10.3390/antiox13091057/s1, Table S1: Sequences used in experiments; Table S2: Antibodies and kits.
