# Supplementary material for: Sodium Houttuybonate Promotes the Browning of White Adipose Tissue by Inhibiting Ferroptosis via the AMPK-NRF2-HO1 Pathway
Source: Antioxidants (Basel). 2024 Aug 30;13(9):1057. doi: 10.3390/antiox13091057 (PMC11428211; doi:10.3390/antiox13091057)
Supplement: Supplementary file 1 [file antioxidants-13-01057-s001.zip › Table S1.pdf]

Table S1. Sequences used in experiments.

Target	Organism	Forward (5'-3')	Reverse (5'-3')
Cidea	Mus musculus	GGTGGACACAGAGGAGTTCTTTC	CGAAGGTGACTCTGGCTATTCC
Prdm16	Mus musculus	ATCCACAGCACGGTGAAGCCAT	ACATCTGCCACAGTCCTTGCA
Ucp1	Mus musculus	AGGCTTCCAGTACCATTAGGT	CTGAGTGAGGCAAAGCTGATTT
Cpt1	Mus musculus	GGTCTTCTCGGGTCGAAAGC	TCCTCCCACCAGTCACTCAC
Cpt2	Mus musculus	GATGGCTGAGTGCTCCAAATACC	GCTGCCAGATACCGTAGAGCAA
Hsl	Mus musculus	GATTTACGCACGATGACACAGT	ACCTGCAAAGACATTAGACAGC
Mgl	Mus musculus	CGGACTTCCAAGTTTTTGTCAGA	GCAGCCACTAGGATGGAGATG
Atgl	Mus musculus	GGATGGCGGCATTTCAGACA	CAAAGGGTTGGGTTGGTTCAG
Cox7a1	Mus musculus	AAACCGTGTGGCAGAGAGAGCAG	CCCAAGCAGTATAGCAGTAGGC
Cox8b	Mus musculus	TGTGGGGATCTCAGCCATAGT	AGTGGGCTAAGACCCATCCTG
Cycs	Mus musculus	CCATCTACACAGAAGTCTTGGAG	GCGTTTTCGATGGTCATGCTCTG
Nrf2	Mus musculus	CAGCATAGAGCAGGACATGGAG	GAACAGCGGTAGTATCAGCCAG
18S	Mus musculus	CGCCATGTCTCTAGTGATCC	GGTCGATGTCTGCTTTCCTC
