# Supplementary material for: Sodium Houttuybonate Promotes the Browning of White Adipose Tissue by Inhibiting Ferroptosis via the AMPK-NRF2-HO1 Pathway
Source: Antioxidants (Basel). 2024 Aug 30;13(9):1057. doi: 10.3390/antiox13091057 (PMC11428211; doi:10.3390/antiox13091057)
Supplement: Supplementary file 1 [file antioxidants-13-01057-s001.zip › Table S2.pdf]

Table S2: Antibodies and kits

Name	Supplier	Cat no.
Triglyceride assay kit	Nanjing Jiancheng	Cat# A110-1-1
Alanine aminotransferase Assay Kit	Nanjing Jiancheng	Cat# C009-2-1
Aspartate aminotransferase Assay Kit	Nanjing Jiancheng	Cat# C010-2-1
Lipid Peroxidation MDA Assay Kit	Beyotime	Cat# S0131S
Ematoxylin-Eosin Stain	aladdin	Cat# E489517
Sodium Houttuyfonate	TargetMol	Cat# 83766-73-8
ML385	TargetMol	Cat# 846557-71-9
TRIzol Reagent	ThermoFisher	Cat#15596018
2 x SYBR® Green Pro Taq HS Premix （Rox Plus）	AG	Cat#AG11718
5 x Evo M-MLV RT Master Mix *1	AG	Cat#AG11706
Collagenase, Type 2	Diamond	Cat#A004174-0001
Dispase® II	Sigma-Aldrich	Cat#D4693-1G
UCP1 Polyclonal antibody	proteintech	Cat# 23673-1-AP
NRF2 Rabbit pAb	Abclonal	Cat#A0674
anti-mouse HSP90	Proteintech	Cat# 60318-1-Ig
anti-mouse P-AMPK	Abclonal	Cat# AP1441
anti-mouse AMPK	Abclonal	Cat# A12718
goat anti-rabbit secondary antibody	Abcam	ab6721
